# Metasurface-Enhanced Antennas for Microwave Brain Imaging

**DOI:** 10.3390/diagnostics11030424

**Published:** 2021-03-03

**Authors:** Eleonora Razzicchia, Pan Lu, Wei Guo, Olympia Karadima, Ioannis Sotiriou, Navid Ghavami, Efthymios Kallos, George Palikaras, Panagiotis Kosmas

**Affiliations:** 1 Faculty of Natural and Mathematical Sciences, King’s College London, Strand, London WC2R 2LS, UK; pan.lu@kcl.ac.uk (P.L.); wei.guo@kcl.ac.uk (W.G.); olympia.karadima@kcl.ac.uk (O.K.); ioannis.sotiriou@kcl.ac.uk (I.S.); navid.ghavami@kcl.ac.uk (N.G.); 2 Metamaterial Inc., Dartmouth, NS B2Y 4M9, Canada; themos.kallos@mediwise.co.uk (E.K.); george.palikaras@metamaterial.com (G.P.)

**Keywords:** metasurface (MTS), enhanced-antenna, microwave tomography (MWT), microwave imaging (MWI), brain imaging

## Abstract

Stroke is a very frequent disorder and one of the major leading causes of death and disability worldwide. Timely detection of stroke is essential in order to select and perform the correct treatment strategy. Thus, the use of an efficient imaging method for an early diagnosis of this syndrome could result in an increased survival’s rate. Nowadays, microwave imaging (MWI) for brain stroke detection and classification has attracted growing interest due to its non-invasive and non-ionising properties. In this paper, we present a feasibility study with the goal of enhancing MWI for stroke detection using metasurface (MTS) loaded antennas. In particular, three MTS-enhanced antennas integrated in different brain scanners are presented. For the first two antennas, which operate in a coupling medium, we show experimental measurements on an elliptical brain-mimicking gel phantom including cylindrical targets representing the bleeding in haemorrhagic stroke (h-stroke) and the not oxygenated tissue in ischaemic stroke (i-stroke). The reconstructed images and transmission and reflection parameter plots show that the MTS loadings improve the performance of our imaging prototype. Specifically, the signal transmitted across our head model is indeed increased by several dB‘s over the desired frequency range of 0.5–2.0 GHz, and an improvement in the quality of the reconstructed images is shown when the MTS is incorporated in the system. We also present a detailed simulation study on the performance of a new printed square monopole antenna (PSMA) operating in air, enhanced by a MTS superstrate loading. In particular, our previous developed brain scanner operating in an infinite lossy matching medium is compared to two tomographic systems operating in air: an 8-PSMA system and an 8-MTS-enhanced PSMA system. Our results show that our MTS superstrate enhances the antennas’ return loss by around 5 dB and increases the signal difference due to the presence of a blood-mimicking target up to 25 dB, which leads to more accurate reconstructions. In conclusion, MTS structures may be a significant hardware advancement towards the development of functional and ergonomic MWI scanners for stroke detection.

## 1. Introduction

Stroke is a clinical syndrome causing acute dysfunction of a brain area and it is linked to a vascular mechanism resulting in a vessel occlusion or rupture. This definition of stroke refers to ischemic stroke (i-stroke), which involves a central nervous system infarction, but also broadly includes intracerebral hemorrhage (h-stroke) and subarachnoid hemorrhage [1]. Stroke is a very frequent disorder and a major cause of adult disability and death. It is the fourth most common cause of death in the UK, with more than 100,000 cases each year and a cost to society around £26 billion a year [2].

An early diagnosis of stroke is necessary, as the brain loses millions of cells every second after the vessel’s occlusion or rupture and this may result in a permanent damage or even death. Additionally, stroke’s treatments differ based on the stroke type, meaning that an incorrect diagnosis could be lethal for the patient [3]. Thus, a fast access to efficient imaging tools is needed to timely initiate the correct treatment. Also, a strict follow-up in the first days (especially for treated patients) would also be desirable to assess the stroke’s evolution. Currently, stroke detection relies on technologies such as computed tomography (CT), magnetic resonance imaging (MRI) and cerebral angiography. Both CT and MRI are able to confirm the diagnosis and identify the stroke’s site. However, both these tools are not portable and thus cannot be easily used by paramedics at the patient’s bedside or found inside ambulances [4]. Moreover, whereas CT is ionizing and has a limited sensitivity for early ischaemic signs, while MRI exposes the patient to a strong magnetic field and needs more time and patient’s cooperation to be performed [5]. More importantly, MRI is a high cost technology and its availability is limited for emergency imaging.

Microwave imaging (MWI) could be a valid alternative to the current diagnostic methods, as it is not invasive, uses non-ionizing radiations and has a data acquisition time which ranges from milliseconds to a few seconds. In addition, it is a technology which can lead to low-cost and portable brain scanners [6]. This imaging technique is based on the difference in the tissues’ dielectric properties, which results in perturbations of the scattered field. The tissue dielectric contrast can be estimated using radar-based or tomographic reconstruction algorithms that are applied to the acquired data [7]. In particular, in microwave tomography (MWT), it is possible to reconstruct a map of the spatial distribution of the dielectric properties of the region of interest through solving an inverse ill-posed electromagnetic (EM) problem [8,9,10].

A MWI setup consists of several antennas placed around the body to be imaged. Typically, there is a homogeneous matching medium between the antennas and the body. Using a lossy matching medium minimizes reflection and couples the transmitted power to the body, but it also attenuates the useful signal transmitted into the body [7]. As a result, the coupling medium affects the detection of useful scattered “weak” signals. As the antenna array that acquires these signals is confined in a small region surrounding the head, small and compact antennas are commonly used [11,12,13]. In addition to small size, MWT antenna arrays for brain imaging must operate in the 0.5–2.0 GHz frequency range to achieve an optimal trade-off between resolution and penetration depth [14,15,16]. Following these requirements, several MWI head scanners including helmets and headbands [17] have been considered.

To couple the transmitted power into the body and improve the detection of useful scattered “weak” signals, we could take advantage of metasurface (MTS) technology. As demonstrated in our previous studies [18,19], one way to improve the efficacy of a MWT system is incorporating MTS structures into its array, adjacent to the substrate of each antenna. Using this hardware configuration, we have obtained higher quality reconstructed images of a blood-mimicking target placed inside the brain volume of our numerical head model. MTS’s are the two-dimensional equivalent of metamaterials (MM’s), which are engineered materials made of sets of small scatterers or apertures arranged in a regular array throughout a region of space. MTS’s are mainly based on sub-wavelength split-ring resonators (SRRs) which can be designed in order to obtain some desirable EM behaviour which is not found in naturally occurring materials, such as negative refractive index or near-zero constitutive parameters [20]. Thus, we can take advantage of MTS structures to manipulate size, efficiency, bandwidth, and directivity of several EM systems [21,22]. For instance, zero-index MM’s (materials with constitutive parameters ϵ’ and μ of zero or near-zero values) and negative index MM’s have shown a strong potential in several applications and can be used to fabricate high directivity antennas [23,24,25]. At microwave frequencies, MTS-enhanced antennas are typically modelled tailoring the MTS’s design in order to interact with the emitted wave to form the desired radiation pattern [26]. Recently, layers of MTS have been used in a variety of clinical applications such as biomedical imaging and sensing. For instance, SRRs have been used to enhance the sensing properties of existing biosensors [27,28]. Likewise, MTS’s have been used as flat lenses for near-field imaging [29] and MRI [30].

With the aim of improving the accuracy of MWI for brain stroke detection and localization, we propose three MTS-enhanced antennas. To this end, this work validates our previous designed MTS film by presenting experiments with an elliptical brain phantom including cylindrical stroke-mimicking targets. Our experimental results suggest that the MTS film employed to enhance the sensitivity of our custom-made MWT prototype [31] can have a positive impact when placed on the head, closely fixed to the MWI array’s antennas. Then, we show a detailed simulation study on the performance of a new MTS-enhanced printed square monopole antenna (PSMA) operating in air. Our results show that we can detect a blood-mimicking target placed inside the brain volume of our head model avoiding the use of a thick and bulky matching medium. In addition, the MTS superstrate loading enhances the dielectric contrast between the blood-mimicking target and the surrounding tissue.

The remainder of the paper is organized as follows: Section 2 presents the methodology for the MTS-enhanced antenna designs. It also describes the geometry of the custom-made prototype used to validate our previous MTS designs and the simulation setups used to test the MTS-enhanced PSMA. Section 3 presents reflection and transmission plots from experiments and images reconstructed through our previously developed 2D DBIM-TwIST algorithm. It also includes simulation results which suggest that the PSMA’s MTS loading increases the MWI system’s sensitivity to the signal scattered by a blood-mimicking target. Finally, Section 4 includes a discussion, concluding remarks and future work.

## 2. Materials and Methods

### 2.1. MTS-Enhanced Antennas Immersed in a Coupling Medium

**Figure 1:**
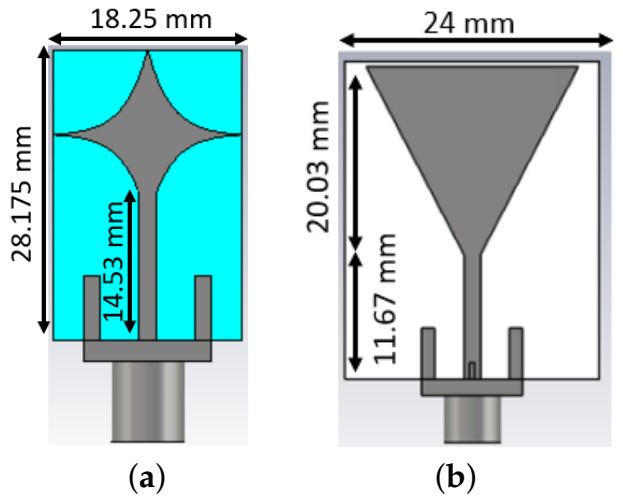
Dimensions of the substrate, patch and transmission line of (**a**) the spear patch antenna and (**b**) the triangular patch antenna.

The triangular patch antenna and the spear patch antenna used in our experiments are shown in Figure 1a,b. These printed monopole antennas were modelled on an FR-4 substrate with a partial ground on the back side. To couple energy more efficiently into the head, both the antennas were designed to operate in a matching medium made of a 90% glycerol-water mixture [17,32,33]. The dimensions of the substrate, patch and transmission line of the antennas are shown in Figure 1a,b.

**Figure 2:**
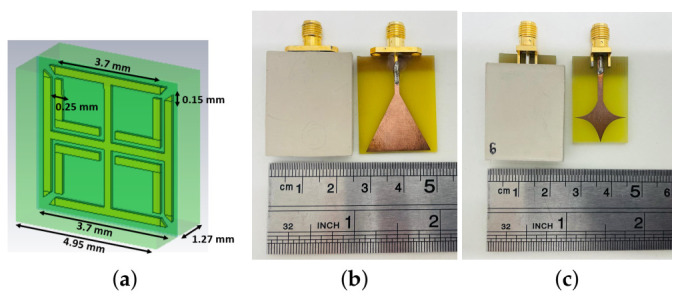
(**a**) Dimensions of the MTS unit cell (**b**) Photograph of the triangular patch antenna and its MTS-enhanced version (**c**) Photograph of the spear patch antenna and its MTS-enhanced version.

A 24.75 mm × 29.7 mm MTS superstrate loading was modelled to operate as an enhancer of the antennas described above and glued on the antennas’ radiating elements. This MTS structure is based on the unit cell geometry shown in Figure 2a, which comprises a Jerusalem Cross-shaped copper lattice (thickness = 0.10 mm) embedded between two Rogers 3010 TM substrates (thickness = 1.27 mm, ϵ = 10.2 and tanδ = 0.0022). These two high dielectric substrates are bonded with with Rogers 3001 bonding film (ϵ = 2.28, tanδ = 0.003). In previous work [18,34], we have performed several simulation studies to optimize this geometry for operating in contact with the human skin when immersed in our coupling medium.

### 2.2. MTS-Enhanced Antennas: Experimental Validation in a Coupling Medium

**Figure 3:**
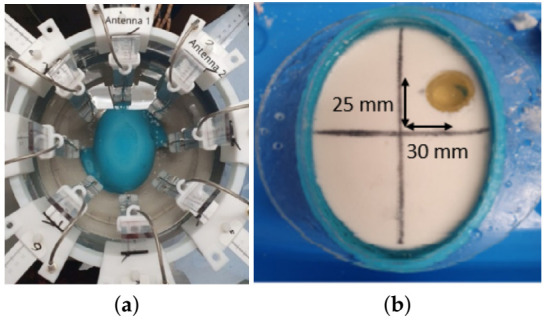
(**a**) Custom-made tomographic experimental setup (**b**) Elliptical mould containing the average brain phantom with the blood-mimicking target.

To test the MTS-enhanced antennas operating in our coupling medium, we carried out experiments using the custom-made MWT prototype shown in Figure 3a. This setup includes an elliptical array placed inside a 300 mm diameter cylindrical tank and connected to a multiport Keysight M9019A Vector Network Analyser (VNA). Measurements were performed by immersing eight transceivers inside the tank, in a 90% glycerol solution used as matching medium. The antennas were placed as close as possible to the phantom’s external surface. To adjust the antennas’ position, we used horizontal and vertical mounts. By sequentially transmitting from one antenna and receiving by the others, the eight-antenna array produces a scattering matrix to be fed into our DBIM-TwIST algorithm for image reconstructions (see Appendix A).

To examine the performance of the MTS-enhanced triangular patch antenna, two gelatin-oil phantoms mimicking the properties of average brain and blood tissue were fabricated, as described in [35]. A 3D printed elliptic mould made of ABS was used as a holder for our brain liquid phantom that, once solidified, can mimic the brain. We first measured the average brain phantom (“no target” scenario) using both the triangular patch antennas and MTS-enhanced antennas. Then, we inserted a 30 mm diameter cylindrical inclusion resembling blood tissue into the brain-mimicking mixture and performed the “with target” measurements.

To test the MTS-enhanced spear antenna, we fabricated new gelatin-oil phantoms mimicking the properties of average brain, blood tissue and ischaemia. After carrying on the “no target” measurements, we inserted the 30 mm inclusion resembling the bleeding in the brain phantom and we performed the “with target” measurements. Then, we substituted the blood-mimicking inclusion with the ischaemia-mimicking target and we carried out a second round of “with target” measurements.

All the inclusions were positioned at the same coordinates, at an angular position of about 320 degrees relative to the position of the first transmitting antenna, as shown in Figure 3b. The permittivity at 1 GHz of the coupling liquid and each phantom used in these experiments is shown in Table 1.

### 2.3. MTS-Enhanced Printed Square Monopole Antenna Operating in Air

**Figure 4:**
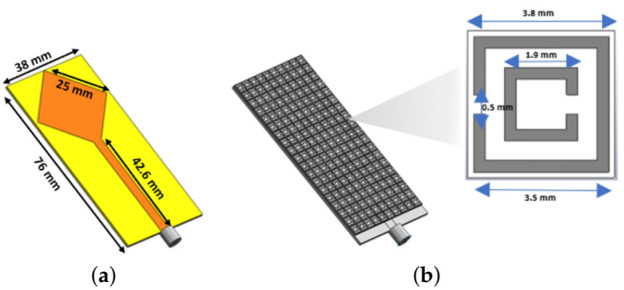
(**a**) Dimensions of PSMA (**b**) PSMA with MTS superstrate loading and square DS-SRR based unit cell.

To investigate the possibility of applying MWT without employing any matching media in between the antennas and the human head, we modelled the printed square monopole antenna (PSMA) shown in Figure 4a using CST Microwave Studio. The proposed PSMA is operating in air and is specifically designed to work in close contact with our numerical head phantom. It is designed on an RT/duroid 5880 LZ low dielectric substrate (thickness = 1.026 mm, ϵ’ = 1.96, tanδ = 0.0019) and is based on a square patch with a 10 mm partial ground on the back side.

After assessing the PSMA’s performance, we designed the MTS-enhanced PSMA shown in Figure 3b. This MTS-enhanced antenna includes a 10×19 unit cell MTS film covering almost the whole antenna’s surface and bonded to the front side of the PSMA through Rogers 3001 bonding film (thickness = 0.03 mm). The MTS unit cell design is based on a 0.01 mm thick Double Square Split-Ring Resonator (DS-SRR) printed on a square RT/duroid 5880 LZ (thickness = 0.635 mm).The dimensions of the DS-SRR are shown in Figure 4b.

**Figure 5:**
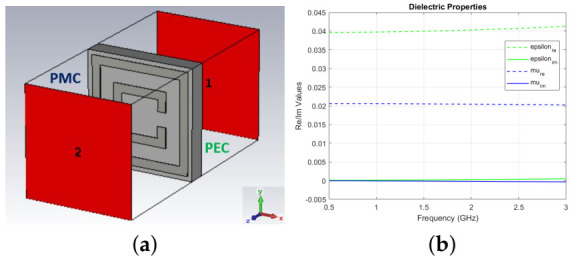
(**a**) Waveguide setup, with an incident wave propagating along Z-direction (**b**) Real and imaginary values of permittivity and permeability for the MTS.

The waveguide setup shown in Figure 5a was used to characterize the proposed MTS unit cell structure. The left and right surfaces are assigned as PEC walls, whereas the top and bottom sides (perpendicular to the two waveguide’s ports) are PMC walls. Transmission and reflection were calculated through CST Microwave Studio. The material properties extracted from the S-Parameters are shown in Figure 5.

### 2.4. PSMA and MTS-Enhanced PSMA: Comparison between Different MWT Scanners

**Figure 6:**
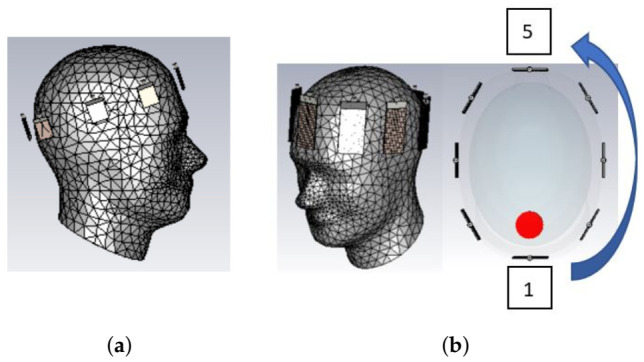
(**a**) 8-PSMA array (“System 1”) (**b**) 8-MTS-Enhanced PSMA array (“System 3”) and its transversal view. “System 2” is equal to “System 2” except for including non-enhanced PSMAs.

To test the performance of our new PSMA and MTS-enhanced PSMA operating in air, we compared our previous developed brain scanner operating in an infinite lossy matching medium to two tomographic systems: an 8-PSMA system and an 8-MTS-enhanced PSMA system. Our previous developed MWT scanner for brain imaging consists of 12 spear patch antennas immersed in a 90% glycerol-water mixture and placed around EN 50361 Specific Anthropomorphic Mannequin (SAM) head model [18,19]. A similar setup (“System 1”), including 8 antennas placed elliptically around our numerical head model, was modelled in CST Microwave Studio. The antenna array was immersed in an infinite mixture of 90% glycerol-water matching medium. Using the same head model, which is made of a nylon mould (ϵ’ = 3.2, tanδ = 0.013) containing an average brain numerical phantom inside (ϵ’ = 45.8 and σ = 0.76 S/m), we studied other two MWT scanners operating in air: “System 2”, which comprises 8 PSMA, and “System 3”, which includes 8 MTS-enhanced PSMA. In these MWT systems, the antennas were closely fixed to the head’s surface. The MWT antenna arrays described above are shown in Figure 6.

For each antenna array, the S-Parameters were measured over the 0.5–2.0 GHz frequency range for the “no target” configuration (head model including average brain only) and the “with target” configuration. This last configuration includes a cylindrical blood-mimicking target (30 mm diameter and 35 mm height) inserted inside the brain volume and placed in the back side of the head model, close to antenna 1. Then, the signal difference “with target–no target” (dB) at relevant frequencies was calculated. Finally, images were reconstructed at the single frequencies 0.9 GHz, 1.0 GHz and 1.1 GHz, by applying our 2D DBIM-TwIST algorithm [36] to the simulated data.

## 3. Results

### 3.1. Experimental Results for the MTS-Enhanced Antennas Operating in a Coupling Medium

In our previous study [19], we have already shown the benefits of using MTS-enhanced antennas. In this section, we present experimental results for both the arrays described in Section 2.2. The reconstructed images and transmission and reflection parameter plots suggest that the MTS superstrate loading illustrated in Figure 2 has the potential to improve the performance of our custom-made tomographic system. The S-Parameter plots are shown for the “no target” and “with target” configuration, with and without MTS superstrate loading. Moreover, to provide more complete information on the enhancement of the system response in the presence of the MTS, we also present field amplitude distributions from accurate numerical simulations of our system interacting with the brain phantom in Section 3.3.

#### 3.1.1. 8-MTS-Enhanced Triangular Patch Antenna Array

**Figure 7:**
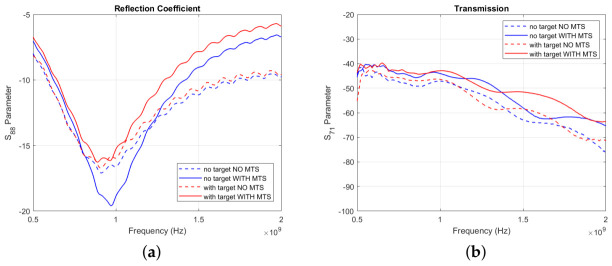
(**a**) Measured $S_{88}$ Parameter for the triangular patch antenna array in the “with target” (red lines) and “no target” (blue lines) configuration, with (solid lines) and without MTS (dashed lines) (**b**) Measured $S_{71}$ Parameter for the triangular patch antenna array in the “with target” (red lines) and “no target” (blue lines) configuration, with (solid lines) and without MTS (dashed lines).

Figure 7a shows the reflection coefficient for one of the triangular patch antennas (number 8 in Figure 6b), which is placed in front of the blood-mimicking target, while Figure 7b shows an example of transmission signal levels (in dB) across the brain phantom, along the direction containing the target. The antenna’s reflection coefficient in the “no target” configuration is reduced by around 3 dB, suggesting that the antenna’s matching is enhanced at the operating frequency when the MTS is present. Moreover, the plotted transmission parameter is improved of around 8 dB over all the considered frequency range.

**Figure 8:**
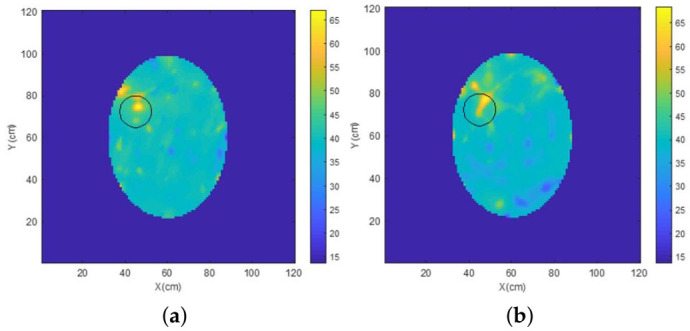
Frequency hopping reconstructions of the blood-mimicking target obtained from experimental data for the triangular patch antenna array (**a**) without MTS and (**b**) with MTS.

Two-dimensional (2D) multi-frequency (frequency hopping) reconstructions were performed through our DBIM-TwIST algorithm [36], which is discussed in detail in Appendix A. The reconstruction results of the dielectric contrast between the blood-mimicking target and the surrounding brain tissue are shown in Figure 8, where three frequencies (0.7 GHz, 1.1 GHz and 1.3 GHz) were used. An improvement in the quality of the reconstructed images is shown when the MTS layers are added to each antenna element. In particular, a reduction of artefacts and a better localization of the blood target are observed.

#### 3.1.2. 8-MTS-Enhanced Spear Patch Antenna Array

**Figure 9:**
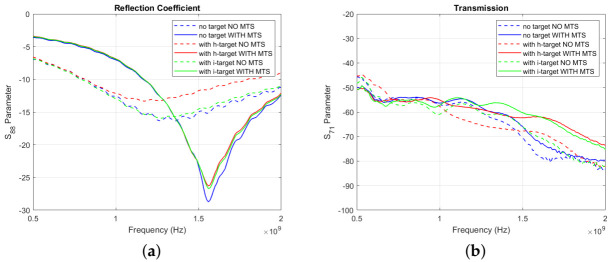
(**a**) Measured $S_{88}$ Parameter for the spear patch antenna array in the “with target” (red lines) and “no target” (blue lines) configuration, with (solid lines) and without MTS (dashed lines) (**b**) Measured $S_{71}$ Parameter for the spear patch antenna array in the “with target” (red lines) and “no target” (blue lines) configuration, with (solid lines) and without MTS (dashed lines).

Figure 9a plots the reflection coefficient vs. frequency for the spear patch antenna number 8, while Figure 9b plots the transmission coefficient vs. frequency for a pair of antennas opposite the brain phantom along the direction containing the haemorrhage-mimicking or ischaemic-mimicking target. These plots suggest that similar improvement in performance is achieved if the MTS is used with the spear antennas, similar to the case of the triangular antennas.

**Figure 10:**
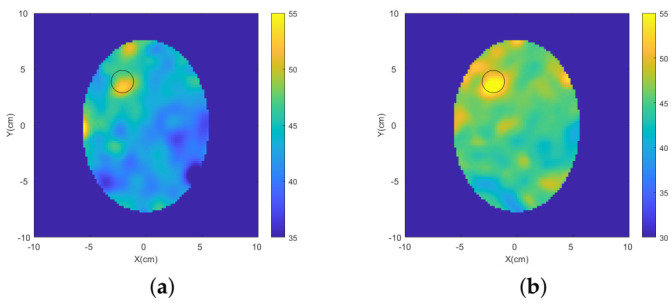
Frequency hopping reconstructions of the blood-mimicking target obtained from experimental data for the spear patch antenna array (**a**) without MTS and (**b**) with MTS.

**Figure 11:**
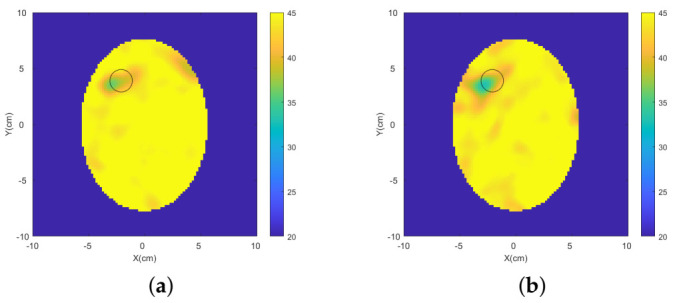
Frequency hopping reconstructions of the ischaemia-mimicking target obtained from experimental data for the spear patch antenna array (**a**) without MTS and (**b**) with MTS.

The blood-mimicking target and the ischaemia-mimicking target were reconstructed via our 2D DBIM-TWiST algorithm. Multi-frequency reconstructions were performed using frequencies 0.7 GHz, 1.1 GHz and 1.3 GHz. The reconstructed blood phantom’s permittivity and ischaemia-mimicking tissue’s permittivity are shown in Figure 10 and Figure 11, respectively. A better localization of the targets and a reduction of the artefacts are shown when the MTS films are integrated in the setup.

### 3.2. PSMA and MTS-Enhanced PSMA: Simulation Results with Different Brain Scanners

**Figure 12:**
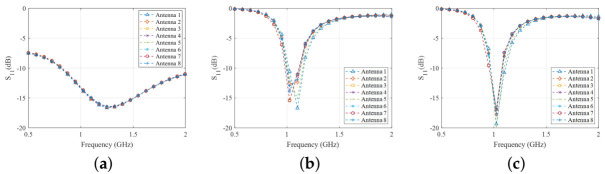
Calculated reflection coefficients in the “no target” scenario for (**a**) the spear patch antennas, (**b**) the PSMAs and (**c**) the MTS-enhanced PSMAs.

For each of the systems described in Section 2.4, the S-Parameters were calculated through CST Microwave Studio over the 0.5–2.0 GHz frequency range. Then, the signal difference “with target - no target” (dB) at relevant frequencies was plotted. Figure 12 shows the reflection parameter for each of the antennas of “System 1”, “System 2” and “System 3”. The plots show that presence of the MTS superstrate loading improves the reflection coefficient of the PSMA by 5 dB.

**Figure 13:**
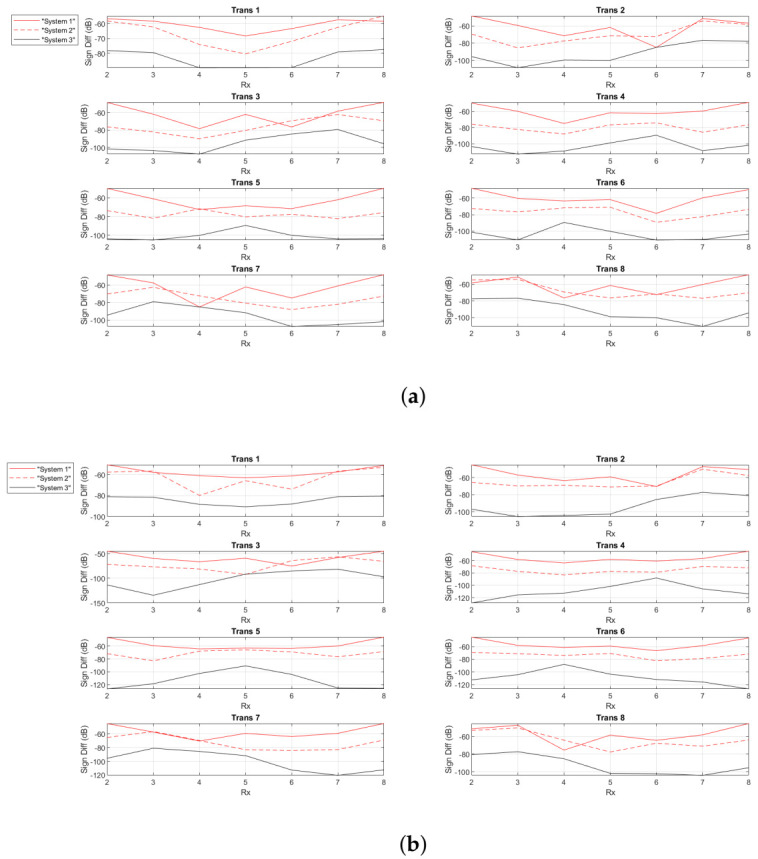
Calculated signal difference “with target - no target” (dB) for the 8-spear patch antenna array operating in infinite matching medium (“System 1”), the 8-PSMA array (“System 2”) and the 8-MTS-enhanced PSMA array (“System 3”) at (**a**) 1 GHz and (**b**) 1.1 GHz.

Figure 13 shows the signal difference “with target – no target” (dB) as a function of receiver location at frequencies 1 GHz and 1.1 GHz for “System 1”, “System 2” and “System 3”. As shown in the graphs, when the PSMA is loaded with our MTS superstrate, it leads to an overall improvement of the signal difference (up to 25 dB) compared to “System 2”. Also, the coupling liquid affects the “weak” signal scattered from the blood-mimicking inclusion, which falls below the noise level.

**Figure 14:**
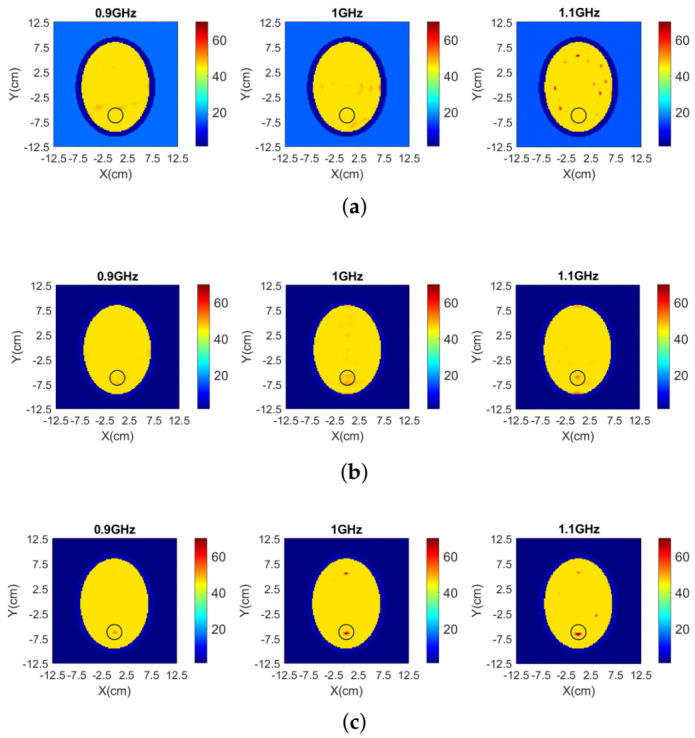
Real part of permittivity reconstructed from simulation data at frequencies 0.9 GHz, 1 GHz and 1.1 GHz for (**a**) the 8-spear patch antenna array operating in infinite matching medium (“System 1”) (**b**) the 8-PSMA array (“System 2”) and (**c**) the 8-MTS-enhanced PSMA array (“System 3”) operating in air.

Finally, to test the MTS’s impact on tomographic reconstructions, we have applied our 2D DBIM-TwIST algorithm to the simulated data plotted in Figure 13. We carried out single-frequency reconstructions, assuming approximate knowledge of the dielectric properties of plastic and average brain tissue as initial guess for our algorithm. The estimated dielectric properties at frequencies 0.9 GHz, 1 GHz and 1.1 GHz is shown in Figure 14. When employing our new PSMA operating in air (“System 2”), the blood-mimicking target is detected correctly. However, a higher contrast between the haemorrhage-mimicking target and the surrounding brain tissue is observed when the MTS is integrated in the scanner (“System 3”).

### 3.3. Near-Field Analysis of the MTS-Enhanced Antennas

**Figure 15:**
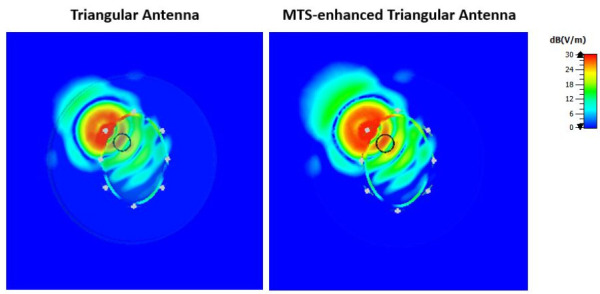
Real part of the E-field calculated at 0.9 GHz and plotted on a transverse section of the simulated brain phantom including a blood-mimicking target. A higher field intensity in the target region is shown when the MTS is placed adjacent on the triangular antenna’s substrate (right plot). In particular, the E-Field values calculated at the centre of the target are 23.14 dB (V/m) and 27.60 dB (V/m) for the triangular patch antenna and the MTS-enhanced triangular patch antenna, respectively.

**Figure 16:**
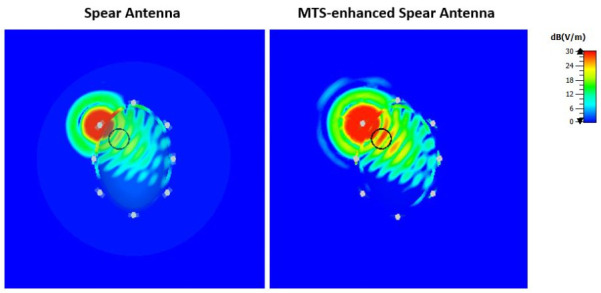
Real part of the E-field calculated at 1.55 GHz and plotted on a transverse section of the simulated brain phantom including a blood-mimicking target. A higher field intensity in the target region is shown when the MTS is placed adjacent on the spear antenna’s substrate (right plot). In particular, the E-Field values calculated at the centre of the target are 20.90 dB (V/m) and 24.90 dB (V/m) for the spear patch antenna and the MTS-enhanced spear patch antenna, respectively.

In near-field MWI, spatial distributions of the electric field magnitude are important measures of antenna performance, which is affected by the impact of evanescent k-space contributions [37]. To evaluate performance in the near field, we have used our simulation setup to calculate examples of spatial distributions due to the transmitting antenna with and without the MTS loading. Examples of these distributions for the real part of the electric field calculated at the antennas’ resonant frequencies are shown in Figure 15 and Figure 16 for the triangular patch antenna and the spear patch antenna, respectively. These results confirm that the antenna-radiated probing fields are significantly stronger in the presence of the MTS superstrate loading, leading to an increased sensitivity of our MWI system.

## 4. Discussion

In this paper, we have presented experiments including brain tissue-mimicking phantoms and cylindrical targets mimicking strokes as well as numerical simulations with several brain scanners. Our study demonstrates that MTS structures have the potential of improving brain imaging when integrated in MWT scanners. The MTS unit cell shown in Figure 2a and the MTS superstrate loadings based on this design were studied experimentally using the custom-made prototype described in Section 2.2. The MTS loadings were shown to enhance transmission and improve the matching of our in-house fabricated antennas in the 0.5–2.0 GHz frequency range. In particular, the signal transmitted across the head phantom, along the direction containing the blood-mimicking target, is overall increased over all the frequency range. Moreover, the antenna reflection coefficient is reduced by several dB when the MTS films are incorporated into the system. This improvement in the near-field antennas performance translates into higher-quality reconstruction images.

Furthermore, an innovative MTS-enhanced antenna design was proposed to test our 2D DBMI-TwIST algorithm in air. To this end, three MWT scanners were tested and compared using a numerical head model made of average brain tissue and including a blood-mimicking target. For each system, the S-Parameters were measured over the 0.5–2.0 GHz frequency range and the signal difference “with target–no target” (dB) at relevant frequencies was calculated. Single frequency reconstructions were also performed. Our results indicate that, when our PSMA is loaded with the MTS superstrate, the signal difference due to the presence of the target is increased up to 25 dB. This translates into a higher contrast between the target and the surrounding tissue. Another interesting point is to note that the signal scattered by the blood-mimicking inclusion is severely affected by the coupling medium. Thus, it is possible to detect a haemorrhage-mimicking target placed inside the brain volume of our numerical head model without using a bulky matching liquid.

To the best of our knowledge, this is the first study presenting experimental and simulation results which demonstrate the possibility of enhancing the detection of stroke-mimicking targets by incorporating MTS antenna enhancers for different MWT arrays. While previous work by various research groups has argued that MWT is applicable for brain imaging (e.g., [38]), improving the sensitivity and hence the quality of the data is critical to achieve clinical accuracy. This feasibility study suggests that MTS technology might be an important step towards the goal of developing functional, portable, and ergonomic MWI scanners with the desired clinical accuracy.

It is of course important to note that most clinical MWI applications involve a three-dimensional (3D) non-linear inverse problem, which is complicated due to the complex structure of the brain and the high dielectric contrast between its different tissues [39]. Thus, in addition to optimising the data acquisition system, the ability of the DBIM-TwIST algorithm to tackle this highly non-linear problem within the distorted Born approximation must be demonstrated. Our previous work has shown that the algorithm can recover complex structures of high dielectric contrast in realistic numerical breast phantoms [36], and some initial simulation results with complex brain phantoms have also shown promise [40]. These studies have shown the importance of the initial guess and prior information as well as frequency hopping in getting the DBIM-TwIST algorithm to converge to meaningful reconstructions of these complex structures.

Our vision of developing a portable microwave stroke detection scanner must of course tackle several additional challenges. These include the requirement to place antenna sensors conformally to the head surface, uncertainty and variability in the properties of the skin-hair-scalp interface, and inhomogeneities and variability in the dielectric properties of brain tissues (including the presence of blood vessels), which are lossy and result in compromising imaging resolution to ensure sufficient penetration. These result in a non-linear, very challenging 3D EM inverse scattering problem, which will certainly require some prior information on the brain tissues distribution and properties. Regardless of the challenges in microwave stroke detection, we must emphasise, however, that our results suggest that MTS-enhanced arrays can be advantageous for various other medical MWI applications (e.g., breast cancer detection) and algorithms (e.g., radar-based methods).

Based on these considerations, our future work will focus on conducting more experimental studies using more complex setups. Regarding the antenna arrays immersed in the coupling liquid, we aim to validate our MTS design using the 3D version of our tomographic algorithm, by employing 24-antenna arrays distributed in two or three rows around the head phantom. Then, we will work towards optimizing the MTS-enhanced PSMA and carrying out experimental studies to assess the feasibility of applying our algorithm without employing any matching media. Before evaluating our MTS-enhanced system with clinical data, we also plan to fabricate and test our system with more realistic inhomogeneous brain phantoms. In particular, we will use digital volumetric brain phantoms with complex structure and different tissues (e.g., grey matter, white matter, bone, muscle and skin) to test the capabilities and requirements of our DBIM-TwIST approach.

## Figures and Tables

**Table 1:** Permittivity of glycerol solution, brain phantom, blood-mimicking target and ischaemia-mimicking target measured at 1 GHz.

Type of Material	Permittivity at 1 GHz
90% Glycerol-water Mixture	15
Average Brain Phantom	38
Blood Phantom	65
Ischaemia Phantom	22

## Abbreviations

The following abbreviations are used in this manuscript:

MWI	Microwave Imaging
MTS	Metasurface
PSMA	Printed Square Monopole Antenna
CT	Computed Tomography
MRI	Magnetic Resonance Imaging
MWT	Microwave Tomography
EM	Electromagnetic
SRR	Split-Ring Resonator
VNA	Vector Network Analyzer
DS-SRR	Double-Square-Split-Ring Resonator
PEC	Perfect Electric Conductor
PMC	Perfect Magnetic Conductor
SAM	Specific Anthropomorphic Mannequin

## Appendix A. The DBIM-TwIST Algorithm

The distorted Born iterative method (DBIM) is an iterative algorithm for solving the electromagnetic (EM) inverse scattering problem, which is used for for medical imaging applications to estimate the spatial distribution of the tissue’s dielectric properties inside a region *V* in the human body (see, for example [41]). The nonlinear inverse scattering problems for each transmitter-receiver (TR) pair is linearized and approximated by the following equation,

(A1) $$\begin{array}{c} E_{s}\left(r_{n},r_{m}\right)=E_{t}\left(r_{n},r_{m}\right)-E_{b}\left(r_{n},r_{m}\right)=\omega^{2}\mu_{0}\epsilon_{0}\int_{V}G_{b}\left(r_{n},r\right)E_{b}\left(r,r_{m}\right)\delta_{\epsilon }\left(r\right)dr, \end{array}$$

where $E_{t}$, $E_{s}$, $E_{b}$ are the total, scattered and background electric fields respectively, $r_{n}$ and $r_{m}$ are the transmitting and receiving antenna locations, ω is the angular frequency, $\mu_{0}$ is the permeability of free space, $\epsilon_{0}$ is the permittivity of free space, and $G_{b}$ is the Green’s function for the background medium. As we only consider point sources, the Green’s functions can be calculated from the electric field as, $G_{b}\left(r_{n},r\right)=\frac{j}{\omega \mu_{0}J}E_{b}\left(r,r_{n}\right)$. The difference $\delta_{\epsilon }$ between the relative complex permittivity of the reconstructed region, $\epsilon_{r}\left(r\right)$, and the background medium, $\epsilon_{b}\left(r\right)$, is defined as $\delta_{\epsilon }\left(r\right)=\epsilon_{r}\left(r\right)-\epsilon_{b}\left(r\right)$.We note that the scalar integral equation above assumes two-dimensional (2D) TM propagation, and is only an approximation of the three-dimensional (3D) inverse problem at hand. Despite this loss in information, the 2D approximation can produce images of acceptable quality in many microwave imaging problems arising in medical applications.

At each DBIM iteration *i*, the integral equation can be discretized for each TR pair as,

(A2) $$E_{s}\left(r_{n},r_{m}\right)=j\omega \epsilon_{0}\sum \delta_{\epsilon }\left(r\right)E_{b}\left(r_{n},r\right)E_{b}\left(r,r_{m}\right),$$

leading to an ill-posed linear system as,

(A3) $$\begin{array}{c} A\delta_{\epsilon }=b \end{array}$$

where A is an M×N matrix (M≪N), with *M* transmit-receive pairs and *N* voxels of the reconstruction region *V*, *b* is a M×1 vector of the scattered fields. The A is calculated at each DBIM iteration by the forward solver which yields $E_{b}$ for a known background $\epsilon_{b}$. The background field is used to build the linear system above, which is then solved by an inverse solver. Finally, the background profile is updated by $\epsilon_{b}^{i+1}\left(r\right)=\epsilon_{b}^{i}\left(r\right)+\delta_{\epsilon }\left(r\right)$ and the DBIM continues to next iteration i+1.

The forward solver of our DBIM-TwIST algorithm uses the finite difference time domain (FDTD) method. The FDTD method simulates the EM wave propagation of the direct, “forward” problem based on Maxwell’s equations. As mentioned above, this work considers only 2D transverse magnetic (TM) waves to reconstruct 2D geometries. Furthermore, our FDTD implementation uses a single-pole Debye model to model frequency-dependent materials such as brain tissues as, $\frac{\varepsilon }{\varepsilon_{0}}=\varepsilon_{\infty }+\frac{\Delta \varepsilon }{1+j\omega \tau }+\frac{\sigma_{s}}{j\omega \varepsilon_{0}}$.

We employ the two-step iterative shrinkage/thresholding (TwIST) algorithm as the solver of the ill-posed linear inverse problem at each DBIM iteration. Thresholding algorithms solve the ill-conditioned linear system Ax=b, by finding a solution *x* which minimizes the least squares error function as $F\left(x\right)=\frac{1}{2}{∥Ax-b∥}^{2}+\lambda {∥x∥}_{1}$ with a regularization term ${\lambda ∥x∥}_{1}$ to stabilize the solution by limiting its $l_{1}$-norm. The general structure of the TwIST algorithm for solving this minimization problem is given by [42],

(A4) $$\begin{array}{cc} \; & x_{t+1}=\left(1-\alpha \right)x_{t-1}+\left(\alpha -\beta \right)x_{t}+{\beta C}_{\lambda }\left(x_{t}\right)\\ & C_{\lambda }\left(x\right)=\Psi_{\lambda }\left(x+A^{T}\left(y-Ax\right)\right) \end{array}$$

The parameters for the TwIST algorithm are calculated as $\kappa =\frac{\xi_{1}}{\xi_{m}},\;\rho =\frac{1-\sqrt{\kappa }}{1+\sqrt{\kappa }},\;\alpha =\rho^{2}+1,\;\beta =\frac{2\alpha }{\xi_{1}+\xi_{m}}$, where $\xi_{1}$ and $\xi_{m}$ are the smallest and largest eigenvalues of $A^{T}A$ respectively. The shrinkage/thresholding operation is a soft-thresholding function, calculated as $\Psi_{\lambda }\left(x\right)=sign\left(x\right)\max\left\{0,|x|-\lambda \right\}$. At each TwIST step, the new solution is updated based on two previous solutions and the soft-thresholding function. As the linear system can be extremely ill-conditioned for microwave imaging problems, the TwIST parameters above must be optimised specifically for the problem at hand [43].

The stopping criterion of the TwIST algorithm can be set based on a tolerance value, which is the normalised difference between the previous and current values of F(x) and is defined as $tol=\frac{F\left(x_{k+1}\right)-F\left(x_{k}\right)}{F(x_{k})}$. The TwIST algorithm stops when tol is smaller than a preset value, usually in range between $10^{-4}$ and $10^{-1}$. This early termination of the iterative algorithm serves an additional regulariser, with a similar effect to a Tikhonov approach [41]. Our $l_{1}$-problem can be regularised further by employing the Pareto curve method, which which yields the optimal tradeoff between the residual error ${∥Ax-b∥}^{2}$ and the norm of the solution ${∥x∥}_{1}$ (similar to the L-curve method for $l_{2}$-problems) [36]. To reduce the computational cost, a practical approach for selecting λ is based on the form $\lambda =\delta ∥A^{T}{b∥}_{\infty }$, where δ is factor with 0<δ<1 [36].
